# Delayed Subaponeurotic Fluid Collection in a Neonate: A Case Report

**DOI:** 10.7759/cureus.58754

**Published:** 2024-04-22

**Authors:** Mohammed Y Abusaleem, Waeel O Hamouda, Marwa K Abdelshafy, Ahmed A Farag, Ashraf I Serhan

**Affiliations:** 1 Pediatrics and Neonatology, Security Forces Hospital Dammam, Dammam, SAU; 2 Neurosurgery, Faculty of Medicine, Teaching, and Research Hospitals, Cairo University, Cairo, EGY; 3 Neurosurgery, Security Forces Hospital Dammam, Dammam, SAU; 4 Pediatrics and Neonatology, National Research Center, Cairo, EGY; 5 Neurosurgery, King Abdullah Medical City, Makkah, SAU

**Keywords:** delayed, neonate, collection, fluid, subaponeurotic

## Abstract

Delayed subaponeurotic fluid collection (DSFC) is a rare cause of scalp swelling that typically presents in healthy-term babies during the second month of life. It is a benign, self-limited condition that requires only conservative management. We present a case of DSFC in a male infant who was brought to our emergency department by his parents at the age of 52 days because of concerns about a newly noticed fluctuating scalp mass. The baby was managed conservatively, and the DSFC completely resolved after three weeks. We describe and discuss the diagnostic workup conducted and the management plan implemented in line with the scientific literature and similar cases previously reported. Given its benign but rare nature, pediatricians and neurosurgeons should be more aware of DSFC as a potential entity in the differential diagnosis of fluctuant scalp swellings. Early recognition can prevent unnecessary investigations or interventions and provide reassurance to parents regarding the condition's benign course. To the best of our knowledge, this is the first reported case in Saudi Arabia, the second reported case from the Middle East, and the second from Asia.

## Introduction

Scalp swelling in the neonatal period is a common clinical condition, and the differential diagnosis must include the most common ones such as cephalohematoma, caput succedaneum, subaponeurotic hemorrhage, and leptomeningeal cyst [1].

Delayed subaponeurotic fluid collection (DSFC) is another differential diagnosis that has been scarcely reported in case reports and case series, and the number of cases in the literature remains few [2]. The natural history of DSFC compared to cephalohematoma and caput succedaneum differs significantly and is important to recognize. DSFC is usually clinically obvious, often reported in the weeks following delivery, and resolves spontaneously with conservative management [3].

We aim to draw attention to DSFC, a clinical condition that represents a diagnostic challenge for most physicians because of its rarity.

## Case presentation

We report a case of a 52-day-old baby boy who was delivered by cesarean section at 39 weeks due to failure to progress. It is important to note that there were no preceding attempts of vaginal delivery, no need for instrumental assistance, and no placement of scalp electrodes during delivery.

Upon birth, the baby had APGAR scores of 7 and 8 at one and five minutes, respectively. His birth weight was 3.1 kg, and his head circumference was 35 cm (within normal +1 standard deviation SD for age). After receiving routine care in the nursery, he was discharged home after 24 hours.

This baby is the first child of his 29-year-old mother. The mother was Rubella immune with negative serologies. She had a normal antenatal history and received routine prenatal care. The parents are not related, and there is no significant family medical history.

**Figure 1:**
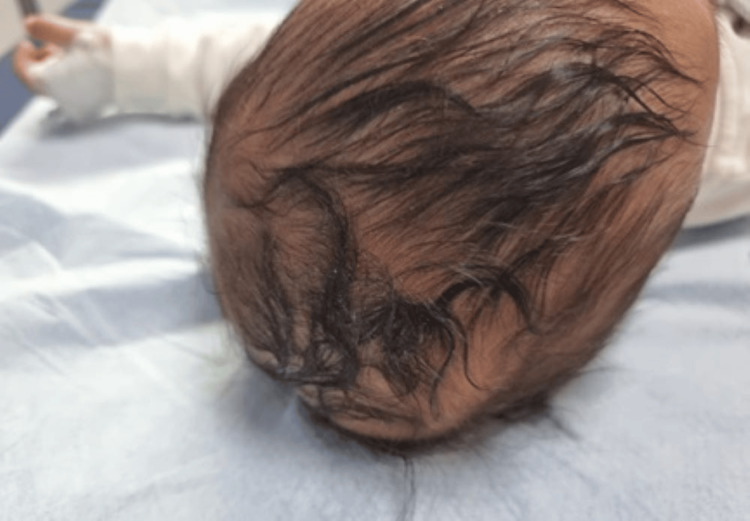
The right posterior parietal soft scalp swelling as presented in the emergency department at 52 days of age.

At 52 days of age, the baby’s parents brought him to our emergency department with a five-day history of new-onset scalp swelling. There was no history of head trauma or fever. Upon examination, the child appeared healthy, active, and alert with stable vital signs. His head circumference measured 38 cm. A soft and fluctuant scalp swelling of approximately 5 cm in diameter was noted on the right posterior parietal area, crossing the posterior part of the sagittal suture (Figure 1).

The swelling was not tender or warm, with no associated bruises, wounds, or changes in the overlying skin. The rest of the systemic examination was unremarkable.

**Figure 2:**
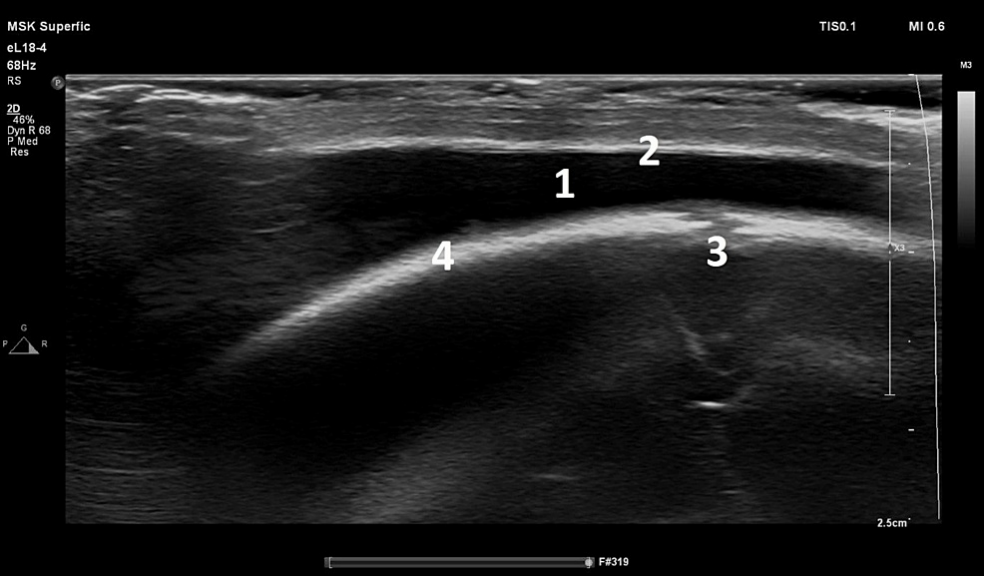
Cranial ultrasonography images Cranial ultrasonography image in coronal orientation done on admission of the case. (1) Subaponeurotic fluid collection with cerebrospinal fluid (CSF)-like hypodensity. (2) Galea aponeorotica layer of the occipitofrontalis muscle appearing as a thin hyperdense scalp layer. (3) The isodense irregular fibrous sagittal suture line marking the midline of the skull between the two pariteal bones. (4) The right parietal bone as a hyperdense structure following the curvature of the skull clavaria.

**Figure 3:**
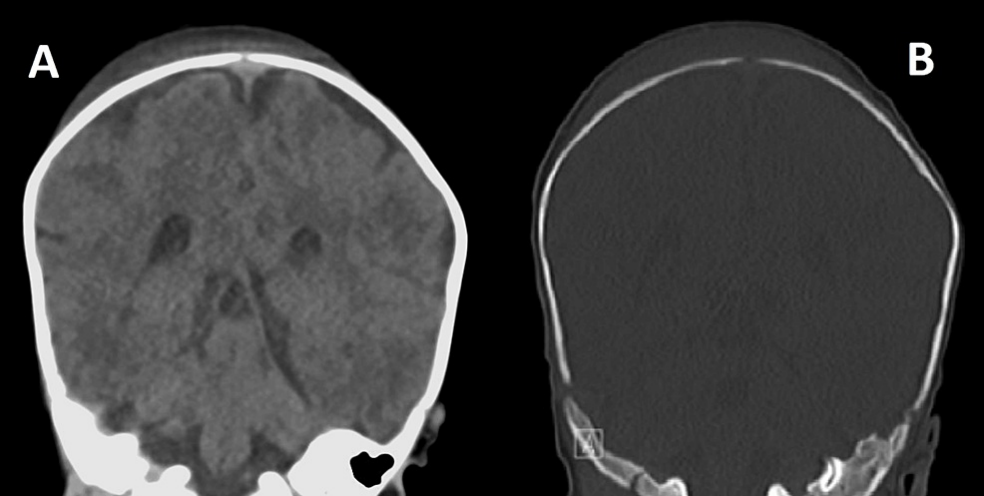
CT brain images of coronal orientation done on admission of the case. A (left image) soft tissue image showing a subaponeurotic scalp swelling crossing the sagittal suture line with no associated intracranial pathology underneath. B (right image) Bone window of the same image showing no detectable skull fractures related to the scalp swelling.

Cranial ultrasonography revealed a hypodense fluid collection in the sub-aponeurotic space measuring approximately 8.5 mm in thickness, which extended across the sagittal suture line (Figure 2). Non-contrast computed tomography (CT) of the brain confirmed a subaponeurotic fluid collection with low Hounsfield density consistent with cerebrospinal fluid (CSF). No skull fractures or other cranial pathologies were detected (Figure 3).

The diagnosis of DSFC was made, and the parents were reassured about the benign nature and course of the condition. The baby was followed up weekly on an outpatient basis, and the swelling completely resolved at 73 days of age (three weeks after initial presentation).

## Discussion

Common causes of scalp swelling in neonates and infants include caput succedaneum, cephalhematoma, and subgaleal hemorrhage. Those lesions typically appear at or shortly after birth, each with its own diagnostic characteristics and management protocols.

Despite being a rare condition, DSFC is yet another cause of scalp swelling in neonates and infants, which may be defined as an extracranial accumulation of fluid occurring between the scalp aponeurosis and the periosteum [1]. This condition usually presents weeks to months after birth, with the mean age of diagnosis being eight weeks and ranging from two to 18 weeks [4]. The etiology is unknown, but it is often associated with instrumental delivery such as ventouse or forceps. Various theories have been proposed, including a potential association with birth trauma, instrumental delivery, disruption of scalp lymphatic or venous drainage, or microfractures in the skull bone resulting in CSF leakage [5].

Multiple case reports and series have reported the findings of X-ray, ultrasound, CT, and MRI images for DSFC as unremarkable, apart from the documentation of a fluid collection between the periosteum and the aponeurosis with CSF characteristics [6]. Management has been universally conservative, with only a few cases undergoing diagnostic or therapeutic tapping. However, in these few cases, the fluid reaccumulated prompting a return to conservative management, ultimately resulting in the spontaneous disappearance of the lesion [7].

Hopkins et al. reported six cases of delayed subaponeurotic fluid collection presenting between three and 18 weeks, which were investigated using X-ray and ultrasound. Five cases were managed conservatively, while one case underwent aspiration twice. All cases resolved within two to 24 weeks [3]. Smith et al. reported 11 cases presenting between three and 12 weeks, which were investigated using X-ray, ultrasound, CT, and MRI, and resolved with conservative management within one to 12 weeks [1]. Wang et al. reported nine cases presenting at two to 11 weeks, which underwent X-ray, ultrasound, CT, and MRI, and resolved with conservative management within two to 20 weeks [4].

To the best of our knowledge, this is the first case reported in Saudi Arabia, the second in the Middle East, and the second in Asia.

## Conclusions

Delayed subaponeurotic fluid collection is a rare cause of fluctuant soft scalp swelling in infants, which typically presents at the second month of age. Radiological investigations are usually unremarkable, except for detecting a CSF-density-like subaponeurotic fluid in the absence of any other intra- or extra-cranial pathology. Conservative management is universally successful, with spontaneous resolution of the swelling within an average period of eight weeks. The nature and course of DSFC are benign and self-limited, thus increased awareness among pediatricians and neurosurgeons would enhance early and accurate differentiation from other more morbid causes of fluctuant soft scalp swelling. This would avoid unnecessary excessive investigations or risky interventions and would help to reassure the parents.
